# Long-Term Tai Chi Experience Promotes Emotional Stability and Slows Gray Matter Atrophy for Elders

**DOI:** 10.3389/fpsyg.2019.00091

**Published:** 2019-01-30

**Authors:** Sijia Liu, Lin Li, Zhiyuan Liu, Xiuyan Guo

**Affiliations:** ^1^School of Psychology and Cognitive Science, East China Normal University, Shanghai, China; ^2^National Demonstration Center for Experimental Psychology Education, East China Normal University, Shanghai, China; ^3^Shanghai Key Laboratory of Magnetic Resonance, School of Physics and Materials Science, East China Normal University, Shanghai, China; ^4^School of Psychology, Shaanxi Normal University, Xi’an, China; ^5^Key Laboratory of Brain Functional Genomics, Ministry of Education, Shanghai Key Laboratory of Brain Functional Genomics, School of Psychology and Cognitive Science, East China Normal University, Shanghai, China

**Keywords:** Tai Chi, VBM, thalamus, hippocampus, emotional stability, gray matter volume

## Abstract

Brain adverse structural changes, especially the atrophy of gray matter, are inevitable in aging. Fortunately, the human brain is plastic throughout its entire life. The current cross-section study aimed to investigate whether long-term Tai Chi exercise could slow gray matter atrophy and explore the possible links among gray matter volume (GMV), long-term Tai Chi experience and emotional stability in a sequential risk-taking task by using voxel-based morphometry. Elders with long-term Tai Chi experience and controls, who were matched to Tai Chi group in age, gender, physical activity level, participated in the study. A T1-weighted multiplanar reconstruction sequence was acquired for each participant. Behaviorally, the Tai Chi group showed higher meditation level, stronger emotional stability and less risk-taking tendency in the sequential risk-taking compared to the control group. Moreover, the results revealed that the GMV of the thalamus and hippocampus were larger in the Tai Chi group compared with the control group. Notably, the GMV of the thalamus was positively correlated with both meditation level and emotional stability. The current study suggested the protective role of long-term Tai Chi exercise at slowing gray matter atrophy, improving the emotional stability and achieving successful aging for elders.

## Introduction

One type of the most mentioned brain adverse structural changes is gray matter atrophy, which is considered as inevitable in normal aging (Csernansky et al., 2004; Braak and Braak, 2010; Walhovd et al., 2011; Oh et al., 2014). The atrophy is not invariably global, as age-related structural change is notable for regional differences. For instance, the thalamus and hippocampus were reported as vulnerable regions connected to age-related degenerative atrophy (Golomb et al., 1993; Sullivan et al., 2004; Testa et al., 2004; Apostolova et al., 2012). Many researches revealed that the thalamus and hippocampus are related to affective response regulation as well as play an important and fundamental role in emotional stability (Davidson et al., 2000; Newberg et al., 2001; Newberg and Iversen, 2003). Thus, gray matter atrophy in the thalamus and hippocampus regions might be the challenge for elders to retain emotional stability (Luders et al., 2009). Many experimental and theoretical literatures described that emotional stability is a vital index of successful aging (Brassen et al., 2011; Carstensen et al., 2011; Suri and Gross, 2012). For example, motivational life-span theory declares that keeping emotional stability is a protective strategy to disengage from negative emotion and beneficial for optimizing well-being (Baltes and Baltes, 1990; Carstensen, 2006; Heckhausen et al., 2010). Based on this information, slowing the atrophy of gray matter volume (GMV) in the hippocampus and thalamus might be propitious to elders in order to maintain emotional stability and achieve successful aging.

Growing evidence suggest that the human brain is plastic through its entire life (Gauthier et al., 2008; Luders et al., 2015). So, it is possible for elders to look for an effective health-management intervention to slow the atrophy of GMV, especially in the thalamus and hippocampus regions. Previous studies revealed that meditation, a mind–body exercise, is beneficial for emotional stability (Tang et al., 2015). Meanwhile, meditation is also considered to be a positive approach to remolding brain structure (Vestergaardpoulsen et al., 2009; Grant et al., 2010; Murakami et al., 2012; Kang et al., 2013; Leung et al., 2013). For instance, Ortner et al. (2007) found that meditators showed reduced emotional responsiveness to unpleasant situation, suggesting an enhanced emotional regulation to avoid potentially harmful effects of negative emotions. Moreover, Hölzel et al. (2008) found that meditators showed greater gray matter concentration in the hippocampus. Consistent with Holzel’s discovery, by using standard whole-brain voxel-based morphometry (VBM) (Ashburner and Friston, 2000), Luders et al. (2009, 2013) reported that the GMV of the hippocampus and thalamus were greater in meditators compared to the control group. Researchers consider that larger GMV in the hippocampus and thalamus of meditators could account for their abilities to disengaging from negative emotion, retain emotional stability, and engage in successful aging (Davidson et al., 2000; Newberg and Iversen, 2003). However, literature has yet to provide assessment of emotional stability with GMV measurement in one study, nor direct evidence for the link between higher emotional stability and larger GMV in the hippocampus and thalamus of meditators. Therefore, more work is needed to justify the inference of the relationship between enhanced emotional stability in meditators and larger GMV of the hippocampus and thalamus.

In fact, meditation encompasses a family of complex practices that include mindfulness meditation, yoga and Tai Chi (Tang et al., 2015). Of these practices, Tai Chi is a popular exercise for elders in China that combines Chinese martial arts and meditative movements with a type of yogic relaxation through deep breathing (Sandlund and Norlander, 2000; Wayne and Kaptchuk, 2008). Previous studies suggested that Tai Chi, as a physical exercise, was an effective method not only to improve health fitness, such as neuromuscular functions, cardiorespiratory system and balance control (Li et al., 2004; Tsang and Huichan, 2005), but also to benefit emotional regulation and psychological well-being for elders (Tammy et al., 2010; Nedeljkovic et al., 2012; Wang et al., 2014). Increasing number of researches have proven that the meditative component in Tai Chi has direct benefits on emotional regulation (Wei et al., 2015). For example, by increasing meditation level to decrease depression and achieve emotional stability (Chiesa et al., 2011). However, few studies have ever explored the relationship among Tai Chi exercise, emotional stability and the changes of GMV in brain regions associated with emotion processing, such as hippocampus and thalamus. The current cross-sectional study (containing Tai Chi group and control group) aims to explore this issue.

Using a sequential risk-taking task (Brassen et al., 2012; Liu et al., 2016, 2017; Li et al., 2018) and VBM, in combination with a developed automated parcellation approach (Tu et al., 2008), we explored whether long-term Tai Chi experience could slow gray matter atrophy and the possible links among long-term Tai Chi experience, emotional stability and the GMV of the hippocampus and thalamus. Accordingly, we predicted that compared with the control group, the group with long-term Tai Chi experience would show higher meditation level and stronger emotional stability to objective outcomes during the sequential risk-taking task. Further, we predicted that long-term Tai Chi experience would slow the atrophy of GMV in the thalamus and hippocampus. More specifically, the Tai Chi group would show larger GMV in their thalamus and hippocampus compared to the control group. In addition, the GMV in the thalamus and hippocampus would be associated with both heightened meditation level and stronger emotional stability.

## Materials and Methods

The current cross-sectional study was approved by East China Normal University Committee on Human Research. All participants provided informed consent using forms approved by the committee.

### Participants

Participants included elderly people residing in local communities that were recruited by public announcement, with a total of 31 Tai Chi participants (10 male, aged from 60 to 70, *M* = 64.94, *SD* = 2.37) and 31 control participants (10 male, aged from 60 to 70, *M* = 64.06, *SD* = 3.05). The inclusion criteria were as follows: (1) participated in Tai Chi training for a long time (10 years) or performed other physical exercise without meditation component, such as playing badminton, fishing, and promenade; (2) no diagnosed neurologic or musculoskeletal disease; and (3) right-handed with normal or corrected-to-normal vision. Overall, all participants were independent, mentally health, community-dwelling older adults with no limiting health conditions that would preclude safe participation in the tests and measurements. Due to incompleteness of scales’ data, the remaining 26 Tai Chi participants and 26 control participants entered in the correlation analyses.

### Scales Data Acquisition

Participants were asked to fill out four scales as follows.

#### Beck Depression Inventory

The Beck Depression Inventory (BDI) (Beck et al., 1988) was used to assess the depression level of participants. The BDI included 21 items which was used to measure the symptoms associated with depression. The split-half coefficient of the Chinese version of the BDI was 0.88 and Cronbach’s alpha was 0.89. The BDI and its individual items exhibited good construct and concurrent validities in China.

#### NEO Five-Factor Inventory

The participants’ personality was measured using the NEO Five-Factor Inventory (NEO-FFI) (Mccrae and Costa, 2004), including five core personality traits: neuroticism, extraversion, openness, conscientiousness, and agreeableness. Each dimension consisted of 12 statements. Participants were asked to rate the degree to which they agreed with these statements. Each statement was rated on a five-point scale (1 = completely agree, 5 = completely disagree), yielding a scale score ranging from 12 to 60.

#### Five Facets Mindfulness Questionnaire

The total score of the Five Facets Mindfulness Questionnaire (FFMQ) was used to assess meditation level of the participants (Deng and Xia, 2011). The FFMQ consisted of 39 items that were rated on a five-point Likert-type scale (1 = never or very rarely true, 5 = very often or always true). This scale measures five distinct facets of mindfulness: (1) observing (defined in terms of noticing or attending to internal and external experiences, noticing internal and external stimuli, including sensation, emotion, cognition, visual perception, e.g., I pay attention to sensations, such as the wind in my hair or the sun on my face); (2) describing (defined in terms of labeling internal experiences with words and discriminating emotional experiences, e.g., I can easily put my beliefs, opinions, and expectations into words); (3) acting with awareness (defined in terms of attending to one’s activities of the moment, e.g., reverse-scoring item: I find myself doing things without paying attention); (4) non-judging of inner experience (defined in terms of taking a non-evaluative stance toward thoughts and feelings, e.g., reverse scoring item: I tell myself I shouldn’t be feeling the way that I am feeling), and (5) non-reactivity to inner experience (defined in terms of allowing thoughts and feelings to come and go, without getting caught up in or carried away by them, e.g., When I have distressing thoughts or images, I just notice them and let them go). The Chinese version of the FFMQ had acceptable psychometric properties and was a valid instrument for the assessment of mindfulness (Baer et al., 2006).

#### Mindful Attention Awareness Scale

The Mindful Attention Awareness Scale (MAAS) was a 15-item instrument measuring the general tendency to be attentive to and aware of present-moment experience in daily life (Brown and Ryan, 2003). It had a single factor structure and yielded a single total score. Using a six-point Likert-type scale (almost always to almost never), respondents rated how often they have experiences of acting on automatic pilot, being preoccupied, and not paying attention to the present moment. The MAAS and its individual items were shown to have good construct and concurrent validities in China (Deng et al., 2012).

#### Barratt Impulsiveness Scale

The Barratt Impulsiveness Scale 11-th (BIS-11) is a self-reporting questionnaire designed to assess the general personality trait of impulsivity. The BIS-11 includes 30 items all rated on a five-point Likert-type scale (1 = rarely, 5 = always). This scale measured three factors of impulsivity: (1) motor impulsivity (acting without thinking, e.g., I do things without thinking); (2) cognitive impulsivity (making quick cognitive decisions, e.g., I make up my mind quickly), and (3) non-planning impulsive (a present orientation or a lack of future planning, e.g., I act on the spur of the moment). The BIS-11 scale and its individual items were shown to have good construct and concurrent validities in China (Yao et al., 2007).

### MRI Data Acquisition

High-resolution T1-weighted MRI data was acquired using a 3.0 Tesla Siemens Trio Tim MRI scanner with a 12-channel head coil at the Shanghai Key Laboratory of Magnetic Resonance (East China Normal University, Shanghai, China). Custom-fit foam pads were used to minimize head movement of the subjects. The parameters of the pulse sequence were as follows: high-resolution T1-weighted three-dimensional magnetization-prepared rapid-acquisition gradient-echo pulse sequence, repetition time = 2530 ms, echo time = 2.34 ms, inversion time = 1100 ms, number of slices = 192, sagittal orientation, field of view = 256 mm × 256 mm, matrix size = 256 × 256, and slice thickness = 1 mm.

### Behavioral Data Acquisition

After obtaining the High-resolution T1-weighted MRI data, participants were told to accomplish a sequential risk-taking task (see more details in Brassen et al., 2012; Liu et al., 2018a). They were told that the payment for their participation was determined by their total obtained coins from the task (1 coin for 1 Chinese yuan).

Participants completed 80 trials in the task. In each trial, an array of eight boxes was presented on a computer screen, where seven boxes contained gold coins (gain) and one box contained a devil (loss). The position of the devil was set randomly on each trial. Participants were instructed to open boxes from left to right and to stop whenever they wanted to collect the coins acquired so far in this trial. They had to decide within 2000 ms to either open the next box and continue or stop and collect the gains acquired so far in that trial by pressing the button. If participants opened the box with the devil, the current trial ended and all coins from that trial were lost. If participants stopped and collected coins, the actual position of the devil was revealed, thus indicating how far they could have safely continued. Next, the outcome was presented for 3000 ms which was highlighted by a cyan square (in the case of stopping and collecting the gains) or a red square (in the case of unpacking the devil and losing the gains). Then, participants were asked to rate how they felt for their choice on a nine-point scale from extreme regret (defined as -4) to extreme relief (defined as 4) in 3000 ms.

### Imaging Processing

Image data processing was conducted using Computational Anatomy Toolbox (CAT 12; Structural Brain Mapping Group, Jena University Hospital, Jena, Germany), which is an extension toolbox of the Statistical Parametric Mapping software (SPM12, Wellcome Trust Centre for Neuroimaging, University College London, United Kingdom) in the MatLab environment^1^. T1 images were bias-field corrected, skull-stripped, aligned to a Montreal Neurological Institute standard space (MNI template), and classified as gray matter, white matter, or cerebrospinal fluid, all within the same generative model (Kurth et al., 2015). For more detail, images were initially assessed for scanner artifacts and gross anatomical abnormalities for each subject. Then, the anterior commissure was set as the origin of spatial coordinates along the reoriented anterior–posterior commissure line. In this analysis, a new fully automated default and standardized method was employed to segment the whole brain structural data into white matter, gray matter and cerebrospinal fluid with symmetric tissue probability maps (TPMs), computed by flipping and averaging (Farokhian et al., 2017). Spatial normalization was achieved by applying the high-dimensional DARTEL approach (Ashburner, 2007). After the segmented gray matter was preserved, we performed a check data quality to assess the homogeneity of the gray matter tissues. All these images were corrected for bias and noise to remove intensity non-uniformities and then registered to the Montreal Neurological Institute (MNI) standard space TPMs. The total intracranial volume (TIV) of each subject was calculated and used as a covariate for further statistical analyses. Prior to the statistical analyses, each participant’s modulated and normalized gray matter tissue segments were smoothed with an 8-mm full width at half maximum Gaussian filter.

### Statistical Analyses of the Behavior Data

In order to investigate the group difference in terms of demography, scores of scales, independent samples *t*-tests were employed. Previous researches usually defined the proportion of loss trials in Gain condition as the tendency index of risk taking (Bornovalova et al., 2005; Reddy et al., 2014). The proportion of loss trials referred to the trial number of the loss trials divided by the total number of trials (here is 80). Group difference in the tendency of risk taking was assessed by an independent samples *t*-test. The outcome of each trial included one of the following two: (1) Gain condition, in which participants did not unpack the devil and gained golds in that trial, (2) Loss condition, in which participants unpacked the devil and lost the golds collected in that trial. Then, we informed a combined index, called real gain-percentage (RGP), which was defined as the ratio of the collected gain and the largest possible gain (that is, the total number of boxes before the devil) in a gain trial (Liu et al., 2016, 2017). The RGP value could be considered as the objective outcome which indicating how good an outcome was in Gain condition. The higher the RGP value, the better the outcome was. Then, according to previous work, the Gain trials were divided into three conditions by using the RGP value: (i) Low RGP (bad outcome): 0 < RGP < = 0.6, (ii) Middle RGP (normal outcome): 0.6 < RGP < = 0.8, and (iii) High GRP (good outcome): 0.8 < RGP < = 1. The cut points between conditions were set *post hoc* to ensure there were sufficient numbers of trials in each condition, making the trial numbers in all conditions as similar to each other as possible (Liu et al., 2018b). Furthermore, we conducted regression analyses within each participant. In the regression analyses, RGP value was defined as dependent variable and emotional ratings were defined as independent variable. The regression coefficient (K) of each subject was calculated. The *K*-value of one participant was considered as index of emotional stability. Specifically, lower *K*-value implied stronger emotional stability. Finally, independent samples *t*-test was performed to compare the differences in emotional stability between the two groups.

### Statistical Analyses of the Image Data

In order to explore whether long-term Tai Chi experience would slow the atrophy of GMV, whole-brain statistical analyses were performed in CAT12 using the spatially normalized and smoothed gray matter maps. Group difference in GMV was evaluated by an independent samples *t*-test. In the analysis mentioned above, age, gender, educational years, and TIV were defined as covariates of no interest to correct different brain sizes. All statistical maps were assigned to a voxel-level threshold of *p* < 0.005 (uncorrected), and a cluster-level threshold of *p* < 0.05 [family-wise error (FWE)]. The surviving clusters were reported. *Post hoc* region of interest (ROI) analyses were performed to test and verify whether the GMV of the thalamus and hippocampus (regions from the independent samples *t*-test described above) were related to behavioral results, such as score of scales, emotional stability and tendency of risk taking. Then, two ROIs were defined as spheres with a radius of 6 mm centered at MNI coordinates -17/-15/0 (left thalamus), -21/-9/-17 (left hippocampus) by using MarsBar toolbox in SPM12. Parameter estimates of GMV were extracted from these ROIs and subjected to correlation analyses. Finally, correlation analyses were conducted to explore relationships between GMV and behavioral data in SPSS 20.0 program (SPSS, Inc., Chicago, IL, United States).

## Results

### Behavior Results

#### Demography and Scales Differences Between the Two Groups

There was no significant difference in demography measurements between the Tai Chi group and control group, such as age, years of education, and physical exercise time per day (Table 1). Except for scores of FFMQ and MAAS, no significant difference was found in the scores of other scales between the two groups (Table 1). More specially, compared to control, the Tai Chi group showed a higher score in FFMQ [*t*(50) = 2.11, *p* < 0.05] and MAAS [*t*(50) = 2.15, *p* < 0.05].

**Table 1:** Demographics and Scale data of both groups.

	Tai Chi group	Control group	*t*	*p*
Gender (male/female)	10/21	10/21	0.00	1.00
Age (years)	64.94 ± 2.37	64.06 ± 3.05	1.26	0.21
Education (years)	10.52 ± 1.91	11.03 ± 2.39	0.94	0.35
Physical exercise time per day (minutes)	66.36 ± 21.96 (Tai Chi practice)	60.14 ± 27.16 (Other exercise)	1.01	0.32
Tai Chi practice (years)	9.98 ± 5.16	0	NA	NA
FFMQ	17.16 ± 1.83	16.18 ± 1.51	2.11	0.04^∗^
MAAS	72.54 ± 11.72	64.62 ± 14.75	2.14	0.04^∗^
BDI	4.96 ± 5.36	5.42 ± 4.78	-0.52	0.61
BIS	59.97 ± 6.73	56.24 ± 10.85	0.317	0.75
NEO-FFI				
Neuroticism	27.31 ± 7.62	30.88 ± 6.07	-1.87	0.07
Extraversion	42.19 ± 6.74	41.04 ± 5.01	0.70	0.49
Openness	38.77 ± 5.14	39.54 ± 4.58	-0.57	0.57
Agreeableness	45.12 ± 6.57	44.19 ± 5.58	0.55	0.59
Conscientiousness	48.27 ± 5.65	48.19 ± 5.00	0.05	0.96

*FFMQ, Five Facets Mindfulness Questionnaire; MAAS, Mindful Attention Awareness Scale; BDI, Beck Depression Inventory; BIS, Barratt Impulsiveness Scale; NEO-FFI, NEO Five-Factor Inventory. ^∗^*p* < 0.05.*

#### Emotional Stability Difference Between the Two Groups

**FIGURE 1:**
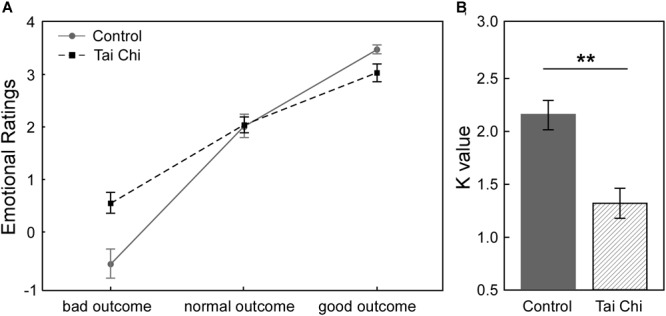
**(A)** In Gain condition, the relationship between subjective emotional ratings and objective outcomes (bad outcome, normal outcome, and good outcome) of two groups. **(B)** The comparison about emotional stability between two groups. The result showed that *K*-value in Tai Chi group was significantly lower than control group [*t*(60) = 3.96, *p* < 0.001]. *K*-value was considered as index of emotional stability. Lower *K*-value reflected stronger emotional stability in Tai Chi group. ^∗∗^*p* < 0.01.

Figure 1A described the relationship between subjective emotional ratings and objective outcomes (bad outcome, normal outcome and good outcome) in the two groups in Gain condition. It showed that participants felt less regret (i.e., more relief) from the bad outcome to the good outcome across all participants. In order to investigate how Tai Chi experience could affect the subjective emotional ratings of outcomes, we supplied the 2 (Group: Tai Chi vs. control) ^∗^ 3 (Outcome: bad outcome, normal outcome, and good outcome) repeated measures ANOVA on emotional ratings. The results revealed significant main effect of Outcome [*F*(2,118) = 203.11, *p* < 0.01] and significant interaction between Group and Outcome [*F*(2,118) = 11.45, *p* < 0.01]. However, no significant main effect of Group was found [*F*(1,59) = 11.45, *p* > 0.05]. Further independent samples *t*-tests revealed that emotional ratings for bad outcome was higher in Tai Chi group than in control group [*t*(60) = 2.772, *p* < 0.01] and emotional ratings for good outcome was lower in Tai Chi group than that in control group [*t*(60) = 2.621, *p* < 0.05]. No significant difference of emotional rating for normal outcome was found between two groups [*t*(60) = 0.218, *p* > 0.05].

Next, to examine whether Tai Chi experience was in favor of enhancing emotional stability, we conducted independent samples *t*-test to compare the difference of emotional stability between the two groups (Figure 1B). The results showed that the *K*-value of the Tai Chi group was lower than that in the control group [*t*(60) = 3.96, *p* < 0.001], indicating stronger emotional stability in the Tai Chi group compared to control group.

#### Tendency of Risk-Taking Difference Between the Two Groups

In order to explore whether there were some differences between the two groups in risk-taking tendency, independent samples *t*-tests were performed. The results showed that the proportion of loss trials in the Tai Chi group was significantly lower than that in control group [Tai Chi group: *M* ±*SD* = 42.7 ± 7.7%; control group: *M* ±*SD* = 51.3 ± 10.1%; *t*(60) = -3.78, *p* < 0.001], indicating that the control group was more risky than the Tai Chi group. Notably, there was no significant difference in the total number of gained coins between the two groups [Tai Chi group: *M* ±*SD* = 164.32 ± 16.28; control group: *M* ±*SD* = 161.65 ± 18.96; *t*(60) = 0.50, *p* > 0.05]. The result suggested that the Tai Chi group could obtain the same number of coins as the control group by using strategies with less tendency of risk-taking.

### VBM Results

#### GMV Differences Between the Two Groups

**FIGURE 2:**
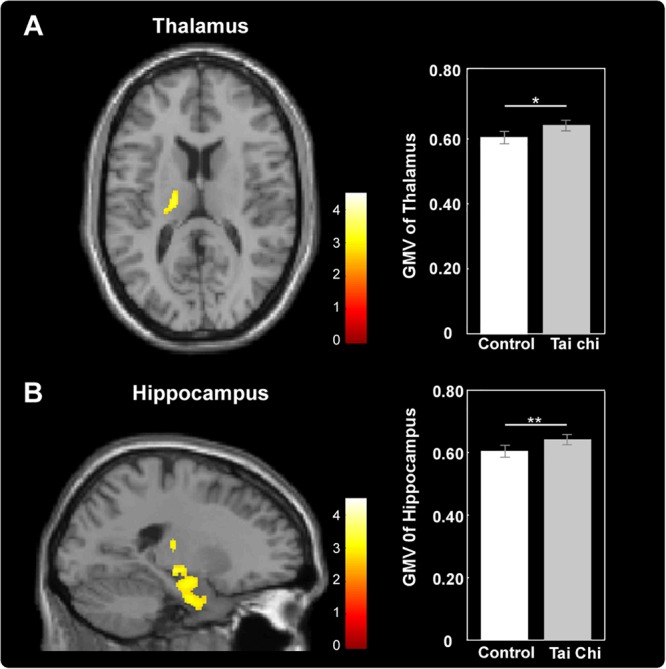
Differences in GMV between Tai Chi group and control group. Tai Chi group showed larger GMV in the left thalamus **(A)** and left hippocampus **(B)**. Cluster level, *p* < 0.05, FWE corrected; voxel level, *p* < 0.005, uncorrected. Error bars indicated one standard error. ^∗^*p* < 0.05, ^∗∗^*p* < 0.01.

Exploratory whole-brain voxel-based independent samples *t*-test analysis was conducted to investigate whether long-term Tai Chi experience slowed the atrophy of GMV. After adjusting for age, gender, educational years and TIV, the comparisons between the two groups showed that the GMV of left thalamus (MNI -17 -15 0) and left hippocampus (MNI -21 -9 -17) were larger in the Tai Chi group than that of control group (Figure 2 and Table 2). There was no significant difference region which revealed larger GMV in the control group than that in the Tai Chi group.

**Table 2:** Anatomical and statistical information of the cluster in which significant between-group differences in gray matter volume (GMV; i.e., Tai Chi group > Control group) were detected.

		MNI coordinates				
Region		*X*	*Y*	*Z*	*t*-Value	Voxels
**Tai Chi > Control**					
L	Parahippocampal gyrus	–24	–9	–30	3.26	1477
L	Hippocampus	–21	–9	–17	3.07	
L	Thalamus	–17	–15	0	2.73	

*Coordinates (mm) are in MNI space. L, left hemisphere; R, right hemisphere. Cluster-level, *p* < 0.05, family-wise error (FWE) corrected, voxel-level, *p* < 0.005, uncorrected.*

### Correlation Analyses

**FIGURE 3:**
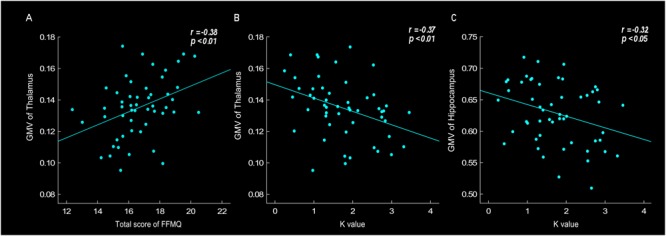
Gray matter volume (GMV) of thalamus was positively correlated with total score of FFMQ (*r* = 0.38, *p* < 0.01) **(A)**, and negatively correlated with *K*-value (*r* = –0.37, *p* < 0.01) **(B)**. GMV of hippocampus was negatively correlated with *K*-value (*r* = –0.32, *p* < 0.05) **(C)**.

Correlation analyses were conducted to investigate the relationship between GMV, especially in the thalamus and hippocampus region, and behavioral data, such as scale’s score, K value and tendency of risk taking. The results found that the thalamus GMV was positively correlated with FFMQ score (*r* = 0.38, *p* < 0.01). Thalamus GMV was also negatively correlated with *K*-value (*r* = -0.37, *p* < 0.01) (Figure 3A,B). The results showed that larger thalamus GMV was related to meditation level and stronger emotional stability. Moreover, the results revealed that the hippocampus GMV was negatively correlated with neuroticism score (*r* = -0.38, *p* < 0.01), *K*-value (Figure 3C, *r* = -0.32, *p* < 0.05) and risk-taking tendency (*r* = -0.35, *p* < 0.05), indicating that larger hippocampus GMV was related to neuroticism level, stronger emotional stability and lower risk-taking propensity.

We further performed a regression analysis to explore the greater contributor to emotional stability. In the regression model, the GMV of the thalamus and hippocampus were defined as predictors and K value was defined as dependent variable. We define their relationship as follows:

$$\begin{array}{c} K{-value = \beta }_{1}{}^{*}{GMV of thalamus + \beta }_{2}{}^{*}{GMV of hippocampus + \beta }_{0} \end{array}$$

The regression coefficients (β_1_ and β_2_) in this model were calculated. The results revealed that the thalamus GMV contributed more to *K*-value than the hippocampus GMV (β_1_ = -2.052, *p* < 0.05; β_2_= -1.407, *p* > 0.05), suggesting that emotional stability variation could be associated with thalamus GMV.

## Discussion

In the current cross-sectional study, we used VBM combined with a sequential risk-taking task to investigate whether long-term (about 10 years) Tai Chi experience could modulate gray matter atrophy and promote emotional stability during the sequential risk-taking task. Consistent with our hypotheses, the results indicated that the Tai Chi group showed improved meditation level and emotional stability compared to the control group. Furthermore, we observed larger GMV in the left thalamus and the left hippocampus of elder people who joined in Tai Chi exercise for an average of 9.98 years, compared with those in the control group. The correlation results also suggested that larger thalamus GMV was associated with higher meditation level as well as stronger emotional stability. In addition, the GMV of the hippocampus was not only positively associated with emotional stability, but also negatively linked with the tendency of risk-taking.

Our behavioral result suggested that the meditation level in the Tai Chi group was significantly higher than the control group, suggesting that Tai Chi exercise might be an effective way to improve meditation level. Researches have revealed that Tai Chi is a multimodal mind-body exercise combing meditation component (Caldwell et al., 2010, 2011). For instance, Caldwell et al. (2010) found that 15-weeks of Tai chi exercise significantly improved meditation level. Moreover, our results also indicated that the Tai Chi group showed stronger emotional stability which might reflected the improved emotion regulation ability in Tai Chi exercisers. Previous research has verified that mindfulness meditation was associated with enhanced ability to regulate emotion (Tang et al., 2015). Because of increased ability of emotional regulation, meditators are associated with less maladaptive emotional regulation, and they are more likely to escape from negative emotions and maintain emotional stability (Ortner et al., 2007). Our results supported that Tai Chi exercise could promote emotional stability just like other types of mindfulness meditation training. Given that, such results might suggest that Tai Chi exercise, that combined a meditation component, played an important role in improving emotional regulation ability.

In the present study, compared with the control group, we observed larger left thalamus GMV in the Tai Chi group after defining age, gender, educational years, and TIV as covariates of no interest. Therefore, it was unlikely that the GMV difference observed in the left thalamus between the two groups was due to demographic characteristics. Our results also revealed that the thalamus GMV was positively related to meditation level and emotional stability, indicating that larger GMV of the thalamus, accompanied with higher level of meditation, might be in favor of emotional regulation. The thalamus is a highly differentiated gray matter structure which governs the flow of sensory information to cortical processing (Newberg and Iversen, 2003), and is characterized as a dynamic conduit and a regulator of the flow of sensory information toward affective situation linking subcortical with cortical areas (Haber and McFarland, 2001). The results in this study paralleled those previously reported in which meditators had larger thalamus GMV in the right or left regions compared to non-meditators. For example, Luders et al. (2009) applied VBM in association with a validated automated parcellation approach and detected significantly larger GMV in the right thalamus of meditators. Moreover, a randomized longitudinal VBM study identified that 8-week meditation training could increase GMV in the region of the left thalamus through exploratory whole brain analysis (Pickut et al., 2013). However, previous studies failed to measure the relationship among thalamus GMV, emotion state and meditation level. Therefore, explanation of enlarged GMV of thalamus in meditators could only be drew from inference based on the function of this brain region. To the best of our knowledge, this is the first imaging study that directly detected the link between the GMV of the thalamus and emotional stability in meditators.

Moreover, the results revealed significantly enlarged left hippocampus GMV in the Tai Chi group compared to control group. This result agreed with previous studies which demonstrated that hippocampus was anatomically altered in meditators (Hölzel et al., 2011b; Eileen et al., 2013; Luders et al., 2013). For example, by using short-term controlled longitudinal study, Hölzel et al. (2011a) reported that after meditation intervention, increased gray matter concentration within the left hippocampus was observed using VBM compared to control group. Luders et al. (2013) examined gray matter characteristics in meditators with long-term experience through cross-section study and found that the GMV of bilateral hippocampus in meditators was larger than control. Accompanied with previous research, our results further indicated that Tai Chi exercise could change the GMV of the left hippocampus similar to mindfulness meditation. In fact, we also found that hippocampus GMV was positively correlated with emotional stability. Previous functional studies verified increased brain activation in the region of hippocampus during meditation by using various imaging methods, such as fMRI (Engström et al., 2010; Monti et al., 2012; Dickenson et al., 2013) and positron emission tomography (PET) (Lou et al., 2015). Davidson et al. (2000) proposed the active role of hippocampus in emotional response to explain activation of hippocampus during meditation. They hypothesized that individuals who habitually failed to regulate their affective responses in a context-sensitive fashion might have a functional impairment of the hippocampus. Therefore, the larger GMV of hippocampus might account for the abilities and habits to maintain emotional stability in Tai Chi exercisers. It was worth noting that regression analysis in the current study indicated, compared to hippocampus GMV, that the thalamus GMV contributed more to emotional stability. As Newberg and Iversen (2003) suggested, the hippocampus could modulate cortical arousal and emotional responsiveness via rich and extensive interconnection with the thalamus. Therefore, our results suggested that the GMV of the thalamus might play a more dominant role in emotional regulation compared to the GMV of the hippocampus.

In addition, as for the relationship between hippocampus GMV and risk-taking tendency, the results displayed that larger GMV of hippocampus was associated with less risk-taking tendency. The hippocampus has been shown as relevant for cognitive and executive functions which might be an explanation of reduced tendency of risk-taking. For instance, Frodl et al. (2006) recruited 34 patients with major depression and 34 healthy controls to investigate whether reduced hippocampus GMV was related to executive dysfunction, by using the Wisconsin Card Sorting Test (WCST). Their results revealed that the GMV of the hippocampus was smaller in depressed patients compared to healthy controls, which was correlated with poorer performance in WCST. They explained that the reduced GMV in the hippocampus might be of considerable functional significance because of its possible relation to impaired executive functions. Building on this, our results showed that larger GMV of the hippocampus was related to rational and cautious decision making. It might suggest that larger GMV of the hippocampus correlates with increased cognitive and executive function to inhibit risk-taking, which is the prospective strategies to optimize successful aging and well-being.

The current study also indicated that the GMV of the hippocampus was negatively correlated with neuroticism score. As neuroticism was closely related to depressive and high-pressure symptom (Bianchi and Laurent, 2016), one possibility was that meditation training enhanced stress resilience, both in terms of subjectively perceived stress as well as objectively measured biomarkers of stress, via similar structural alterations in hippocampus (Jung et al., 2010, 2012; Mohan et al., 2011; Jensen et al., 2012; Fox et al., 2014). Salat et al. (2004) summarized that the hippocampus played a major role in stress and depress regulation. For example, hippocampal atrophy was associated with numerous clinical disorders involving stress, anxiety, and depression (Gurvits et al., 1996; Hölzel et al., 2011a). The current study revealed that Tai Chi could strengthen the ability to disengage from stress states, suggesting it could resist age-related depression, which was the cornerstone to optimize successful aging.

This study has some limitations that should be addressed by future studies. Ideally, individuals in control group should have performed the same physical activities. However, matching participants on this variable proved to be difficult. Given that, the results might not generalize robustly and could be caused by other factors that were not matched and measured. This should be carefully considered when attributing causality. Therefore, in our future studies, we plan to involve longitudinal randomized controlled trials and correlations with other peripheral measures to clearly explore the effects of Tai Chi on the GMV of the thalamus and hippocampus.

## Conclusion

The current study investigated whether long-term Tai Chi experience could slow the atrophy of GMV in the thalamus and hippocampus by using VBM. We explored the relationship among the changes of GMV in brain regions, especially in the thalamus and hippocampus, Tai Chi experience and emotional stability during a sequential risk-taking task. Behaviorally, Tai Chi group showed higher meditation level, stronger emotional stability and decreased tendency of risk-taking compared to the control group. The VBM results showed that the GMV of the left thalamus and left hippocampus were larger in the Tai Chi group compared to the control group. Moreover, the GMV of the thalamus was positively correlated with both meditation level and emotional stability. Our findings highlighted that long-term Tai Chi experience might be beneficial to promote elders’ emotional stability and slow gray matter atrophy in the thalamus and hippocampus. The meditation component of Tai Chi exercise might play a key role in this process.

## Ethics Statement

This study was carried out in accordance with the recommendations of Ethics Committee on Human Experiments of East China Normal University. The protocol was approved by the Ethics Committee on Human Experiments of East China Normal University. All subjects gave written informed consent in accordance with the Declaration of Helsinki.

## Author Contributions

ZL, XG, and LL designed the experiments. ZL programmed the experimental scenario and performed the experiments. ZL and SL analyzed the data. LL, ZL, SL, and XG joined in the interpretation of data. SL, ZL, and LL carried out the writing. All authors read and approved the final version of the manuscript for submission.

## Conflict of Interest Statement

The authors declare that the research was conducted in the absence of any commercial or financial relationships that could be construed as a potential conflict of interest.

## Footnotes

1 www.mathworks.com

## References

[B1] Apostolova L. G. Green A. E. Babakchanian S. Hwang K. S. Chou Y. Y. Toga A. W. (2012). Hippocampal atrophy and ventricular enlargement in normal aging, mild cognitive impairment (MCI), and Alzheimer Disease. *Alzheimer Dis. Assoc. Disord.* 26 17–27. 10.1097/WAD.0b013e3182163b62 22343374 PMC3286134

[B2] Ashburner J. (2007). A fast diffeomorphic image registration algorithm. *Neuroimage* 38 95–113. 10.1016/j.neuroimage.2007.07.007 17761438

[B3] Ashburner J. Friston K. J. (2000). Voxel-based morphometry–the methods. *Neuroimage* 11 805–821. 10.1006/nimg.2000.0582 10860804

[B4] Baer R. A. Smith G. T. Hopkins J. Krietemeyer J. Toney L. (2006). Using self-report assessment methods to explore facets of mindfulness. *Assessment* 13 27–45. 10.1177/1073191105283504 16443717

[B5] Baltes P. B. Baltes M. M. (1990). Successful aging: perspectives from the behavioral sciences. *J. Nerv. Ment. Dis.* 180 1–34.

[B6] Beck A. T. Steer R. A. Garbin M. G. (1988). Psychometric properties of the beck depression inventory: twenty-five years of evaluation. *Clin. Psychol. Rev.* 8 77–100. 10.1016/0272-7358(88)90050-5

[B7] Bianchi R. Laurent E. (2016). Depressive symptomatology should be systematically controlled for in neuroticism research. *Neuroimage* 125 1099–1100. 10.1016/j.neuroimage.2015.07.088 26285078

[B8] Bornovalova M. A. Daughters S. B. Hernandez G. D. Richards J. B. Lejuez C. W. (2005). Differences in impulsivity and risk-taking propensity between primary users of crack cocaine and primary users of heroin in a residential substance-use program. *Exp. Clin. Psychopharmacol.* 13 311–318. 10.1037/1064-1297.13.4.311 16366761

[B9] Braak H. Braak E. (2010). Demonstration of amyloid deposits and neurofibrillary changes in whole brain sections. *Brain Pathol.* 1 213–216. 10.1111/j.1750-3639.1991.tb00661.x 1669710

[B10] Brassen S. Gamer M. Büchel C. (2011). Anterior cingulate activation is related to a positivity bias and emotional stability in successful aging. *Biol. Psychiatry* 70 131–137. 10.1016/j.biopsych.2010.10.013 21183158

[B11] Brassen S. Gamer M. Peters J. Gluth S. Büchel C. (2012). Don’t look back in anger! responsiveness to missed chances in successful and nonsuccessful aging. *Science* 336 612–614. 10.1126/science.1217516 22517323

[B12] Brown K. W. Ryan R. M. (2003). The benefits of being present: mindfulness and its role in psychological well-being. *J. Pers. Soc. Psychol.* 84 822–848. 10.1037/0022-3514.84.4.822 12703651

[B13] Caldwell K. Emery L. Harrison M. Greeson J. (2011). Changes in mindfulness, well-being, and sleep quality in college students through taijiquan courses: a cohort control study. *J. Altern. Complement. Med.* 17 931–938. 10.1089/acm.2010.0645 21999153 PMC3199537

[B14] Caldwell K. Harrison M. Adams M. Quin R. H. Greeson J. (2010). Developing mindfulness in college students through movement-based courses: effects on self-regulatory self-efficacy, mood, stress, and sleep quality. *J. Am. Coll. Health* 58 433–442. 10.1080/07448480903540481 20304755 PMC2879280

[B15] Carstensen L. (2006). The influence of a sense of time on human development. *Science* 312 1913–1915. 10.1126/science.1127488 16809530 PMC2790864

[B16] Carstensen L. L. Turan B. Scheibe S. Ram N. Ersnerhershfield H. Samanezlarkin G. R. (2011). Emotional experience improves with age: evidence based on over 10 years of experience sampling. *Psychol. Aging* 26 21–33. 10.1037/a0021285 20973600 PMC3332527

[B17] Chiesa A. Calati R. Serretti A. (2011). Does mindfulness training improve cognitive abilities? A systematic review of neuropsychological findings. *Clin. Psychol. Rev.* 31 449–464. 10.1016/j.cpr.2010.11.003 21183265

[B18] Csernansky J. G. Hamstra J. Wang L. Mckeel D. Price J. L. Gado M. (2004). Correlations between antemortem hippocampal volume and postmortem neuropathology in AD subjects. *Alzheimer Dis. Assoc. Disord.* 18 190–195. 15592129

[B19] Davidson R. J. Jackson D. C. Kalin N. H. (2000). Emotion, plasticity, context, and regulation: perspectives from affective neuroscience. *Psychol. Bull.* 126 890–909. 10.1037/0033-2909.126.6.890 11107881

[B20] Deng Y. Q. Li S. Tang Y. Y. Ryan R. Brown K. (2012). Psychometric properties of the chinese translation of the mindful attention awareness scale (MAAS). *Mindfulness* 3 10–14. 10.1016/j.aap.2015.04.009 25912099

[B21] Deng Y. Q. Xia C. Y. (2011). The five facet mindfulness questionnaire: psychometric properties of the chinese version. *Mindfulness* 2 123–128. 10.1177/1073191113485121 23596271

[B22] Dickenson J. Berkman E. T. Arch J. Lieberman M. D. (2013). Neural correlates of focused attention during a brief mindfulness induction. *Soc. Cogn. Affect. Neurosci.* 8 40–47. 10.1093/scan/nss030 22383804 PMC3541487

[B23] Eileen L. Florian K. Toga A. W. Narr K. L. Christian G. (2013). Meditation effects within the hippocampal complex revealed by voxel-based morphometry and cytoarchitectonic probabilistic mapping. *Front. Psychol.* 4:398. 10.3389/fpsyg.2013.00398 23847572 PMC3705194

[B24] Engström M. Pihlsgård J. Lundberg P. Söderfeldt B. (2010). Functional magnetic resonance imaging of hippocampal activation during silent mantra meditation. *J. Altern. Complement. Med.* 16 1253–1258. 10.1089/acm.2009.0706 21138386

[B25] Farokhian F. Beheshti I. Sone D. Matsuda H. (2017). Comparing CAT12 and VBM8 for detecting brain morphological abnormalities in temporal lobe epilepsy. *Front. Neurol.* 8:428. 10.3389/fneur.2017.00428 28883807 PMC5573734

[B26] Fox K. C. R. Nijeboer S. Dixon M. L. Floman J. L. Ellamil M. Rumak S. P. (2014). Is meditation associated with altered brain structure? A systematic review and meta-analysis of morphometric neuroimaging in meditation practitioners. *Neurosci. Biobehav. Rev.* 43 48–73. 10.1016/j.neubiorev.2014.03.016 24705269

[B27] Frodl T. Schaub A. Banac S. Charypar M. Jäger M. Kümmler P. (2006). Reduced hippocampal volume correlates with executive dysfunctioning in major depression. *J. Psychiatry Neurosci.* 31 316–325. 16951734 PMC1557684

[B28] Gauthier L. V. Taub E. Perkins C. Ortmann M. Mark V. W. Uswatte G. (2008). Remodeling the brain: plastic structural brain changes produced by different motor therapies after stroke. *Stroke J. Cereb. Circ.* 39 1520–1515. 10.1161/STROKEAHA.107.502229 18323492 PMC2574634

[B29] Golomb J. de Leon M. J. Kluger A. George A. E. Tarshish C. Ferris S. H. (1993). Hippocampal atrophy in normal aging: an association with recent memory impairment. *Arch. Neurol.* 50 967–973. 10.1001/archneur.1993.00540090066012 8363451

[B30] Grant J. A. Courtemanche J. Duerden E. G. Duncan G. H. Rainville P. (2010). Cortical thickness and pain sensitivity in zen meditators. *Emotion* 10 43–53. 10.1037/a0018334 20141301

[B31] Gurvits T. V. Shenton M. E. Hokama H. Ohta H. Lasko N. B. Gilbertson M. W. (1996). Magnetic resonance imaging study of hippocampal volume in chronic, combat-related posttraumatic stress disorder. *Biol. Psychiatry* 40 1091–1099. 10.1016/S0006-3223(96)00229-6 8931911 PMC2910907

[B32] Haber S. McFarland N. (2001). The place of the thalamus in frontal cortical-basal ganglia circuits. *Neuroscientist* 7 315–324. 10.1177/107385840100700408 11488397

[B33] Heckhausen J. Wrosch C. Schulz R. (2010). A motivational theory of life-span development. *Psychol. Rev.* 117 32–60. 10.1037/a0017668 20063963 PMC2820305

[B34] Hölzel B. K. Carmody J. Vangel M. Congleton C. Yerramsetti S. M. Gard T. (2011a). Mindfulness practice leads to increases in regional brain gray matter density. *Psychiatry Res. Neuroimaging* 191 36–43. 10.1016/j.pscychresns.2010.08.006 21071182 PMC3004979

[B35] Hölzel B. K. Lazar S. W. Gard T. Schumanolivier Z. Vago D. R. Ott U. (2011b). How does mindfulness meditation work? proposing mechanisms of action from a conceptual and neural perspective. *Perspect. Psychol. Sci. J. Assoc. Psychol. Sci.* 6 537–559. 10.1177/1745691611419671 26168376

[B36] Hölzel B. K. Ott U. Gard T. Hempel H. Weygandt M. Morgen K. (2008). Investigation of mindfulness meditation practitioners with voxel-based morphometry. *Soc. Cogn. Affect. Neurosci.* 3 55–61. 10.1093/scan/nsm038 19015095 PMC2569815

[B37] Jensen C. G. Vangkilde S. Frokjaer V. Hasselbalch S. G. (2012). Mindfulness training affects attention-or is it attentional effort? *J. Exp. Psychol. Gen.* 141 106–123. 10.1037/a0024931 21910559

[B38] Jung Y. H. Kang D. H. Byun M. S. Shim G. Kwon S. J. Jang G. E. (2012). Influence of brain-derived neurotrophic factor and catechol O-methyl transferase polymorphisms on effects of meditation on plasma catecholamines and stress. *Stress Int. J. Biol. Stress* 15 97–104. 10.3109/10253890.2011.592880 21790467

[B39] Jung Y. H. Kang D. H. Jang J. H. Park H. Y. Byun M. S. Kwon S. J. (2010). The effects of mind-body training on stress reduction, positive affect, and plasma catecholamines. *Neurosci. Lett.* 479 138–142. 10.1016/j.neulet.2010.05.048 20546836

[B40] Kang D. H. Hang J. J. Jung W. H. Sun H. K. Jung Y. H. Choi C. H. (2013). The effect of meditation on brain structure: cortical thickness mapping and diffusion tensor imaging. *Soc. Cogn. Affect. Neurosci.* 8 27–33. 10.1093/scan/nss056 22569185 PMC3541490

[B41] Kurth F. Gaser C. Luders E. (2015). A 12-step user guide for analyzing voxel-wise gray matter asymmetries in statistical parametric mapping (SPM). *Nat. Protoc.* 10 293–304. 10.1038/nprot.2015.014 25591011

[B42] Leung M.-K. Chan C. C. H. Yin J. Lee C.-F. So K.-F. Lee T. M. C. (2013). Increased gray matter volume in the right angular and posterior parahippocampal gyri in loving-kindness meditators. *Soc. Cogn. Affect. Neurosci.* 8 34–39. 10.1093/scan/nss076 22814662 PMC3541494

[B43] Li F. Harmer P. Fisher K. J. Mcauley E. (2004). Tai Chi: improving functional balance and predicting subsequent falls in older persons. *Med. Sci. Sports Exerc.* 36 2046–2052. 10.1249/01.MSS.0000147590.54632.E7 15570138

[B44] Li L. Liu Z. Niu H. Zheng L. Cheng X. Sun P. (2018). Responsibility modulates the neural correlates of regret during the sequential risk-taking task. *Exp. Brain Res.* 236 1–11. 10.1007/s00221-017-5165-3 29299641

[B45] Liu Z. Li L. Li Z. Hu Z. Roberts I. D. Guo X. (2016). The neural basis of regret and relief during a sequential risk-taking task. *Neuroscience* 327 136–145. 10.1016/j.neuroscience.2016.04.018 27102420

[B46] Liu Z. Li L. Zheng L. Xu M. Zhou F. A. Guo X. (2017). Attentional deployment impacts neural response to regret. *Sci. Rep.* 7:41374. 10.1038/srep41374 28145480 PMC5286415

[B47] Liu Z. Wu Y. Li L. Guo X. (2018a). Functional connectivity within the executive control network mediates the effects of long-term tai chi exercise on elders’ emotion regulation. *Front. Aging Neurosci.* 10:315. 10.3389/fnagi.2018.00315 30405392 PMC6205982

[B48] Liu Z. Zheng L. Li L. Xu J. Cheng X. Guo X. (2018b). Social comparison modulates the neural responses to regret and subsequent risk-taking behavior. *Soc. Cogn. Affect. Neurosci.* 13 1059–1070. 10.1093/scan/nsy066 30371903 PMC6204486

[B49] Lou H. C. Kjaer T. W. Friberg L. Wildschiodtz G. Holm S. Nowak M. (2015). A 15O-H2O PET study of meditation and the resting state of normal consciousness. *Hum. Brain Mapp.* 7 98–105. 10.1002/(SICI)1097-0193(1999)7:2<98::AID-HBM3>3.0.CO;2-M 9950067 PMC6873339

[B50] Luders E. Cherbuin N. Kurth F. (2015). Forever Young(er): potential age-defying effects of long-term meditation on gray matter atrophy. *Deutsche Z. Akupunktur* 58 30–31. 10.3389/fpsyg.2014.01551 25653628 PMC4300906

[B51] Luders E. Thompson P. M. Kurth F. Hong J.-Y. Phillips O. R. Wang Y. (2013). Global and regional alterations of hippocampal anatomy in long-term meditation practitioners. *Hum. Brain Mapp.* 34 3369–3375. 10.1002/hbm.22153 22815233 PMC4084509

[B52] Luders E. Toga A. W. Lepore N. Gaser C. (2009). The underlying anatomical correlates of long-term meditation: larger hippocampal and frontal volumes of gray matter. *Neuroimage* 45 672–678. 10.1016/j.neuroimage.2008.12.061 19280691 PMC3184843

[B53] Mccrae R. R. Costa P. T. (2004). A contemplated revision of the NEO Five-Factor Inventory. *Personal. Individ. Differ.* 36 587–596. 10.1016/S0191-8869(03)00118-1

[B54] Mohan A. Sharma R. Bijlani R. L. (2011). Effect of meditation on stress-induced changes in cognitive functions. *J. Altern. Complement. Med.* 17 207–212. 10.1089/acm.2010.0142 21417807

[B55] Monti D. A. Kash K. M. Kunkel E. J. Brainard G. Wintering N. Moss A. S. (2012). Changes in cerebral blood flow and anxiety associated with an 8-week mindfulness programme in women with breast cancer. *Stress Health* 28 397–407. 10.1002/smi.2470 23129559

[B56] Murakami H. Nakao T. Matsunaga M. Kasuya Y. Shinoda J. Yamada J. (2012). The structure of mindful brain. *PLoS One* 7:e46377. 10.1371/journal.pone.0046377 23029500 PMC3460809

[B57] Nedeljkovic M. Ausfeld-Hafter B. Streitberger K. Seiler R. Wirtz P. H. (2012). Taiji practice attenuates psychobiological stress reactivity – a randomized controlled trial in healthy subjects. *Psychoneuroendocrinology* 37 1171–1180. 10.1016/j.psyneuen.2011.12.007 22222120

[B58] Newberg A. Alavi A. Baime M. Pourdehnad M. Santanna J. (2001). The measurement of regional cerebral blood flow during the complex cognitive task of meditation: a preliminary SPECT study. *Psychiatry Res. Neuroimaging* 106 113–122. 10.1016/S0925-4927(01)00074-9 11306250

[B59] Newberg A. B. Iversen J. (2003). The neural basis of the complex mental task of meditation: neurotransmitter and neurochemical considerations. *Med. Hypotheses* 61 282–291. 10.1016/S0306-9877(03)00175-0 12888320

[B60] Oh H. Madison C. Villeneuve S. Markley C. Jagust W. J. (2014). Association of gray matter atrophy with age, β-amyloid, and cognition in aging. *Cereb. Cortex* 24 1609–1618. 10.1093/cercor/bht017 23389995 PMC4014182

[B61] Ortner C. N. M. Kilner S. J. Zelazo P. D. (2007). Mindfulness meditation and reduced emotional interference on a cognitive task. *Mot. Emot.* 31 271–283. 10.1007/s11031-007-9076-7

[B62] Pickut B. A. Van H. W. Kerckhofs E. Mariën P. Vanneste S. Cras P. (2013). Mindfulness based intervention in Parkinson’s disease leads to structural brain changes on MRI: a randomized controlled longitudinal trial. *Clin. Neurol. Neurosurg.* 115 2419–2425. 10.1016/j.clineuro.2013.10.002 24184066

[B63] Reddy L. F. Lee J. Davis M. C. Altshuler L. Glahn D. C. Miklowitz D. J. (2014). Impulsivity and risk taking in bipolar disorder and schizophrenia. *Neuropsychopharmacology* 39 456–463. 10.1038/npp.2013.218 23963117 PMC3870783

[B64] Salat D. H. Buckner R. L. Snyder A. Z. Greve D. N. Desikan R. S. R. Busa E. (2004). Thinning of the cerebral cortex in aging. *Cereb. Cortex* 14 721–730. 10.1093/cercor/bhh032 15054051

[B65] Sandlund E. S. Norlander T. (2000). The effects of Tai Chi Chuan relaxation and exercise on stress responses and well-being: an overview of research. *Int. J. Stress Manag.* 7 139–149. 10.1023/A:1009536319034

[B66] Sullivan E. V. Rosenbloom M. Serventi K. L. Pfefferbaum A. (2004). Effects of age and sex on volumes of the thalamus, pons, and cortex. *Neurobiol. Aging* 25 185–192. 10.1016/S0197-4580(03)00044-7 14749136

[B67] Suri G. Gross J. J. (2012). Emotion regulation and successful aging. *Trends Cogn. Sci.* 16 409–410. 10.1016/j.tics.2012.06.007 22739000

[B68] Tammy S. Bruce K. Judith R. Raveendhara B. Wang C. Schmid C. H. (2010). Tai Chi on psychological well-being: systematic review and meta-analysis. *BMC Complement. Altern. Med.* 10:23 10.1186/1472-6882-10-23 PMC2893078 20492638

[B69] Tang Y. Y. Hölzel B. K. Posner M. I. (2015). The neuroscience of mindfulness meditation. *Nat. Rev. Neurosci.* 16 213–225. 10.1038/nrn3916 25783612

[B70] Testa C. Laakso M. P. Sabattoli F. Rossi R. Beltramello A. Soininen H. (2004). A comparison between the accuracy of voxel-based morphometry and hippocampal volumetry in Alzheimer’s disease. *J. Magn. Reson. Imaging* 19 274–282. 10.1002/jmri.20001 14994294

[B71] Tsang W. W. Huichan C. W. (2005). Comparison of muscle torque, balance, and confidence in older tai chi and healthy adults. *Med. Sci. Sports Exerc.* 37 280–289. 10.1249/01.MSS.0000152735.06282.58 15692325

[B72] Tu Z. Narr K. L. Dollar P. Dinov I. Thompson P. M. Toga A. W. (2008). Brain anatomical structure segmentation by hybrid discriminative/generative models. *IEEE Trans. Med. Imaging* 27 495–508. 10.1109/TMI.2007.908121 18390346 PMC2807446

[B73] Vestergaardpoulsen P. Van B. M. Skewes J. Bjarkam C. R. Stubberup M. Bertelsen J. (2009). Long-term meditation is associated with increased gray matter density in the brain stem. *Neuroreport* 20 170–174. 10.1097/WNR.0b013e328320012a 19104459

[B74] Walhovd K. B. Westlye L. T. Amlien I. Espeseth T. Reinvang I. Raz N. (2011). Consistent neuroanatomical age-related volume differences across multiple samples. *Neurobiol. Aging* 32 916–932. 10.1016/j.neurobiolaging.2009.05.013 19570593 PMC4040218

[B75] Wang F. Lee E.-K. O. Wu T. Benson H. Fricchione G. Wang W. (2014). The effects of tai chi on depression, anxiety, and psychological well-being: a systematic review and meta-analysis. *Int. J. Behav. Med.* 21 605–617. 10.1007/s12529-013-9351-9 24078491

[B76] Wayne P. M. Kaptchuk T. J. (2008). Challenges inherent to t’ai chi research: part I–t’ai chi as a complex multicomponent intervention. *J. Altern. Complement. Med.* 14 95–102. 10.1089/acm.2007.7170A 18199021

[B77] Wei G. X. Dong H. M. Yang Z. Zuo X. N. (2015). Tai Chi Chuan optimizes the functional organization of the intrinsic human brain architecture in older adults. *Sci. Foundation China* 6 54–54. 10.3389/fnagi.2014.00074 24860494 PMC4029006

[B78] Yao S. Yang H. Zhu X. Auerbach R. P. Abela J. R. Pulleyblank R. W. (2007). An examination of the psychometric properties of the Chinese version of the barratt impulsiveness scale, 11th version in a sample of Chinese adolescents. *Percept. Mot. Skills* 104 1169–1182. 10.2466/PMS.104.3.1169-1182 17879649

